# Arenas of Choice During Addiction Treatment: A Qualitative Study of Patient and Staff Experiences

**DOI:** 10.1177/14550725261436983

**Published:** 2026-04-12

**Authors:** Anna Frisint, Christina Andersson, Morten Sager, Lena Eriksson, Annika Jakobsson, Fredrik Spak

**Affiliations:** 1 621628 Department of Health Sciences, Mid Sweden University, Östersund, Sweden; 2 Institute of Neuroscience and Physiology, Occupational Therapy, 70712 University of Gothenburg, Gothenburg, Sweden; 3 Department of Philosophy, Linguistics, and Theory of Science, 334267 University of Gothenburg, Gothenburg, Sweden; 4 Verksamhet Beroende, 56749 Sahlgrenska University Hospital, Gothenburg, Sweden

**Keywords:** addiction treatment, choice, decision-making, exploratory, qualitative, substance dependence

## Abstract

**Background:**

Patient self-determination and integrity are central to quality health care. In addiction treatment, patients encounter choices related to treatment options, yet little is known about how such choices are expressed in practice. The present study aimed to explore how treatment-related choices are expressed and in what situations they occur, from the perspectives of patients and healthcare staff.

**Methods:**

A qualitative exploratory design was applied. Observations and interviews were conducted at a Swedish addiction day care unit over seven weeks. Data were analyzed using content analysis.

**Results:**

Choices emerged throughout the treatment process across different treatment arenas, from initial assessment to discharge. The analysis identified seven subthemes of choice, organized into two themes: (1) unconditional choices reflecting active involvement in treatment (considered choices, spontaneous choices, and delegated choices) and (2) conditional choices, reflecting more passive involvement (negotiated choices, menu choices, either-or choices and hindered choices).

**Conclusions:**

Patients’ choices were shaped by their life circumstances and constrained by staff routines, organizational procedures and treatment policies. Emphasizing patient choice and health literacy during treatment may support informed decision-making and strengthen self-efficacy in maintaining a drug-free life. Future research is recommended to examine the gap between opportunities for choice and treatment routines and policies.

## Introduction

In recent decades, Swedish health care has undergone reforms intended to strengthen patient influence and opportunities for choice. According to the Health Care and Medical Services Act (SFS 2017:30) and the Patient Act (SFS 2014:821), health care should be of good quality, adapted to individual needs, and respect the patient's integrity and self-determination. These principles pose challenges in addiction treatment, where patients are expected to take part in decisions concerning their care, but where the ability and willingness to make such choices may vary. Since choices made in addiction treatment may be life-defining, influencing both immediate recovery and long-term health outcomes, it is important to better understand how opportunities for choice are created and enacted in practice.

Previous research shows both possibilities and limitations in supporting patient choice in addiction care. A systematic review of shared decision-making in substance use disorder treatment (Friedrichs et al., 2016) found that only three of 25 trials showed significant effects when treatments were matched to patients’ preferences. Other studies emphasize the challenge of finding the right timing for patient involvement (Finnell & Lee, 2011; McKay et al., 2015) and of developing novel ways to engage patients in decision-making (Park, 2020). Discrepancies have also been observed between patients’ treatment goals and the outcome measures used in clinical practice (Rosic et al., 2021). Furthermore, not all patients wish to make choices about their treatment, and some regret or want to revise earlier decisions (Jakobsson et al., 2005). This ambivalence occurs against the backdrop of an ongoing debate on whether addiction is best understood as an illness or as a matter of individual choice. While the disease model and the choice model are often presented as opposites, recent scholarship emphasizes processes of choice as central regardless of perspective (Heather and Segal, 2016; Pickard, 2017; Racine & Rousseau-Lesage, 2017).

Theorizing choice in addiction care highlights its complex and situated character. From the perspective of institutional processes, decision-making is embedded in organizational routines and social practices that shape what choices are possible and how they are expressed (Campbell & Gregor, 2008; Mykhalovskiy et al., 2020). This perspective highlights that patients’ opportunities for choice in addiction care are not solely individual or clinical, but also conditioned by broader organizational and social structures. Mol (2008) has critiqued the prevailing “logic of choice”, which frames patients as consumers and care as a product with predefined options, instead proposing that both choice and care emerge through ongoing relational practices. Similarly, Racine and Rousseau-Lesage (2017) argue for a dynamic understanding of voluntary action that acknowledges both internal influences, such as self-control and cognitive strategies, and external influences from context and interpersonal relationships. Hammell (2020) also emphasizes that making choices should be seen as an intersubjective phenomenon, shaped by the interaction between individual capacities and everyday life circumstances. Together, these perspectives problematize individualistic notions of free choice and underscore the importance of examining how choices are negotiated in practice.

Against this background, there is still limited knowledge of how opportunities for choice are expressed in everyday addiction treatment contexts. To support patients’ recovery processes and strengthen motivation for change, it is important to understand not only whether patients can choose, but also how choices unfold in the interaction between patients, staff and institutional frameworks. This study contributes to filling that gap by focusing on a Swedish day care unit (DCU) for tapering addictive substances. The present study aimed to investigate what kinds of choices occur, how they are expressed, and in what situations they take place, as seen from the perspectives of patients and healthcare staff.

## Methods

### Ethical Considerations

The study was granted ethical approval (Dnr. 2020-02195). The participants volunteered to take part in the project after being informed orally and in writing about the aims, methods and planned form for publication. Participants were also informed that, at any time, they could withdraw their consent to participate in a particular part of the study or in total. All participants provided their written informed consent and agreed to maintain integrity and create a respectful atmosphere throughout the data collection process.

### Design

Qualitative methods such as interviews, observations and field studies are recommended for increased understanding of practices and processes, not least in health care (Savage, 2000). Through their presence, researchers gain an insight into the environment and detailed descriptions (Geertz, 1973) of what people actually do, as well as how they interact and talk to each other. In this way, empirical material is created that provides an opportunity to investigate the perspectives that characterize a certain praxis, such as a DCU. Qualitative research in health care has contributed to increasing the understanding of patients’ experiences, but how the organization, structure, policy and guidelines of the care system influence these experiences still remain to be investigated (Rowland et al., 2019).

This study has a qualitative design and an exploratory approach to identify and describe how organizational and institutional factors affect individuals in a care environment (Racine & Barned, 2019; Rowland et al., 2019).

### Participants

The study was carried out at a DCU addiction treatment center in a Swedish University Hospital. Individuals who take addictive substances, legal and illegal, and want outpatient help to phase out use of these substances, can apply to the DCU. Most patients have psychiatric comorbidity, often severe. The patients are offered daytime treatment 5 days a week. The treatment aims to support the patient to achieve their goal to become drug-free and to deal with their psychiatric comorbidity.

The first selection of participants was based on convenience sampling (Morse, 2007). Patients and staff who attended DCU at the time of data collection were asked to participate in the study, and all those who agreed were included. Purposeful sampling (Morse, 2007) was used for the interviews, based on a variety of formal and social positions among the patients and staff. Data were collected through observations of events and conversations with patients and staff. After the data collection had begun, new patients and new staff arrived. They were also informed about the study, and those who were interested were included. In total, 39 individuals participated in the study; 14 were patients and 25 were staff and peer support workers.

Several of the patients had attended the DCU for tapering drugs before this present care session. About half of them were aged less than 40 years and most were men. The professions represented in the department were registered nurses, doctors, assistant nurses, occupational therapists, psychologists, a secretary and unit managers. One day per week, two peer supporters were present. Peer support workers share patients’ experiences of addiction or psychiatric problems and are employed by the organization; they meet patients individually and in groups. As a result of COVID-19, the DCU was obliged to rearrange and reduce both the arrangements and content of care.

### Data

The lead investigator (AF) spent 70 h during 7 weeks in the DCU to collect data through observations and interviews with patients and staff. Data collection lasted for 3–5 h per day, after which time was set aside for writing field notes. A variety of professions, patients and types of formal, informal, individual and group-based conversations were observed. Six interviews were audio recorded, and they varied in length between 17 and 70 min. The observations and interview questions were focused on options for choice and based partly on what emerged in previous observations and interviews and partly on the researchers’ collective knowledge of the social relations that constitute the problem being investigated.

Written documents significant for the research question that included information about how individual activities were coordinated and organized were also collected (Kearney et al., 2019); for example, worksheets from meetings between patients and staff, manuals from group activities, and the University Hospital's routine descriptions.

During the initial analysis, the research group decided to add supplementary data from another week of data collection, focusing on individual meetings (between patients and staff) and another patient interview.

AF was a 39-year-old woman with 7 years of experience as an occupational therapist in psychiatry, but addiction care and the DCU in the study were unfamiliar. AF had experience of participatory observation and individual interviews and was guided during the data collection with continuous supervision by experienced research colleagues. During the initial phase, LE, a researcher from the field of Science and Technology Studies (STS), took part in data collection.

### Data Analysis

**Figure 1.**
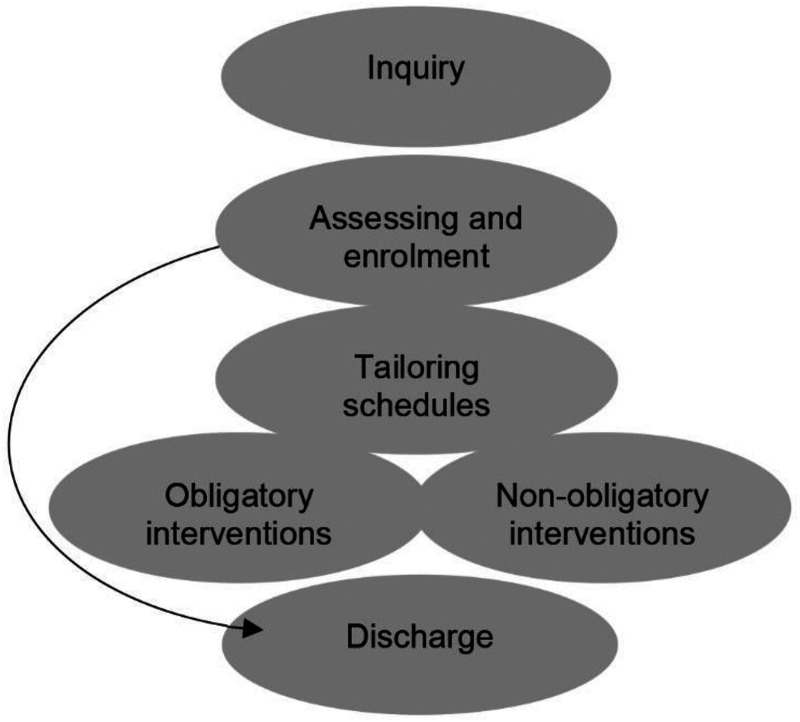
Arenas of choices during patient contact with the day care unit.

The analysis process was iterative and data collection and processing were performed in parallel. Gradual increased understanding of activities comprising choices, such as when, where and how choices emerged at the unit, guided the analysis process. New situations and people helped to create a pattern for the opportunities for choices that occurred. NVivo (https://lumivero.com/products/nvivo) was used for sorting data. The multidisciplinary research group had access to all collected material, and the process was reflective and carried out together. The data were merged and emphasis was placed on mapping how activities including individual choices were expressed and coordinated and could be related to each other. Content analysis (Graneheim & Lundman, 2004) was used for coding all data; written text from observations, interviews, documents and guidelines was read line by line to search for expressions indicating choices made. When such expressions were found, they were marked and the situation and the types of choices were coded with descriptive codes, close to the original text. This led to the organization and description of six arenas of choice (Figure 1). Two themes were identified describing the types of choices that took place (Table 1). The preliminary results were triangulated and confirmed in dialogue with the healthcare staff before setting themes and categories.

**Table 1.** Themes and subthemes of choices identified during the treatment process.

Themes	Choice typology	Definition
Conditional choices showing passive involvement regarding treatment		
Subthemes	Either-or choice*	Only two options, i.e., yes or no, “take it or leave it”, e.g., “if you want to be a patient here, you must follow these rules”
	Menu choice*	Patients making choices based on alternatives presented; the staff present the alternatives and recommend the choices to be made
	Hindered choice	The environment or other external circumstances, such as access to necessary information, limit the possibility of making preferred choices; can be significantly more complicated than an either-or choice
	Negotiated choice	A choice that includes a discussion held between patient and staff, among staff, or as a negotiation with oneself, where conflicting priorities are weighed against each other until a compromise is reached
Unconditional choices showing active involvement regarding treatment		
Subthemes	Spontaneous choice	Made quickly in the moment, sometimes followed by an action; can be more or less reflected upon beforehand, in more or less complex situations
	Delegated choice	An act that involves consciously leaving a choice to be made by another person, profession, or referring to a guideline, e.g., deliberately refraining from the possibility to choose; implies an action, after more or less consideration, leaving the choice to someone or something else
	Considered choice	Encompasses a process whereby pros and cons are considered; includes taking the time to reflect among conceivable alternatives

*Choices made by patients only.

## Results

The results are presented according to the six steps identified in the treatment process and within each step the choices are described. The quotes included in the text are drawn from the interviews and observations reflecting both patients’ and staff's perspectives on the available options. All quotes are anonymous but are labelled to indicate the group affiliation of the respondent.

Opportunities for making decisions appeared in a variety of situations during treatment at the DCU. Some were related to everyday life and others were more obviously linked to the main choice of becoming drug-free or reducing use of addictive substances. In our analysis, we identified seven subthemes of choice, organized into two themes: conditional and unconditional choices (Table 1).

The conditional theme contains four subthemes where reflections are hampered due to external restrictions, regulations or contrasting opinions. When the choices are conditional, more passive behaviors were observed. This theme can be exemplified by the situation where the patient has presupposed wishes for treatment (e.g., keeping some addictive medication despite agreement to taper off others) and this is counteracted by medical restrictions from staff.

In contrast, unconditional choices encompass three subthemes whereby patients and staff can make open choices, more or less independently, considering reflections and needs to a large extent. Furthermore, the unconditional theme entails more activity among those involved. This can be exemplified by the weekly conference where staff discuss different options for patient enrollment, taking into consideration regulations, patient preferences and possibilities for treatment outcomes.

Within the seven subthemes, five subthemes encompassed choices made by both patients and staff. Two subthemes involved patient choices only. All subthemes emerged on various occasions during the treatment process from inquiry to discharge.

A schematic showing the treatment process, identifying six main arenas, is shown in Figure 1. The inquiry marks an institutional starting point where a variety of actors and guidelines contribute to the patients’ choice to seek contact for addiction treatment at the DCU. A choice in one arena can lead to a new situation (and choice) in another arena; in this way, the treatment period includes movement back and forth between choice arenas. For example, tailoring of a patient schedule may lead to a revised choice in the arena for assessing and enrollment, or to a choice to end the treatment period and be discharged. Alas, different choices may have an impact on the main goal for treatment: abstaining from addictive substances.

### Inquiry

Most patients apply via self-referral, which means that they have made a “considered choice” because they realize that they need to phase out the drugs and that they need support (or treatment) to accomplish this goal. When seeking help, patients express that adequate addiction treatment can be difficult to find. When patients choose to seek help from health care, they are depending on others’ knowledge and sometimes on referral to a DCU. Here, patients are sometimes faced with a “hindered choice”, when they experience that information is not available or not applicable for them to make a “considered choice”:

… oh I mean … that general health care, the health center and GPs and so on, they have no knowledge whatsoever about this. (Patient AB)

Staff at the DCU do not have the task of recruiting patients. For further information about the DCU, staff refer patients to the hospital's website, the national information site, with regional relevant information, and to the outpatient clinics. Here, staff make “delegated choices” when informing patients how to access different types of information sources.

In this arena, a tension is shown between organizational guidelines and patients’ wish for expertise. As a result, patients’ opportunities to make choices may be reduced.

### Assessing and Enrollment

Local guidelines state that staff shall assess patients’ suitability for treatment at the DCU. The choice about enrolling a patient is made at a weekly conference, using several available sources, such as referrals, completed assessment interviews, patients’ medical records, and consultations with the patient or staff at another unit where the patient is registered. The assessment of suitable patients is somewhat unclear in guidelines, and the staff handle a complexity of information at these consultation meetings; thereby, the staff are making a “considered choice”. Staff can also be seen to be making a “negotiated choice” when different standpoints about enrollment are discussed. When assessment is made, a staff member contacts the person and offers an enrollment interview. The staff explain that the information the patient receives at the enrollment interview can vary depending on which staff hold the meeting, but also that how the patient interprets the information can vary. Staff make “considered choice” when balancing what and how much information the patient can receive at the enrollment interview. Patients were also seen to be making a “negotiated choice” with themself when uttering ambivalence about tapering off all medication.

Further, the patients who are offered enrollment make an “either-or choice” to accept or reject the offer per se. Most patients express gratitude for being offered treatment at the DCU. To be enrolled, patients are expected to follow established rules, e.g., participate in the daily routines and keep appointments, and avoid taking drugs other than those prescribed. Patients mention concerns about being reprimanded if they fail to keep agreements, e.g., occurrence of lapses and relapses, or fail to adhere to set times in day care and express relief when they are allowed to remain at DCU despite relapses. Staff says that:

…during that journey [treatment period] a lot can happen. It doesn't have to have much to do with the care; things can happen in a person's private or social life that spill over into the care here … but of course we have to reconsider it here, as well as the contract. (Staff Z)

This remark implies that staff makes “considered choices” related to the patients’ overall situation when making decisions about reprimands or ending the treatment session. For the patient, still this leads to another “either-or choice” to accept the rules or give up the treatment.

### Tailored Schedules

According to healthcare regulations, all patients together with staff must establish an individual care plan. The care plan includes the patients’ aim for the treatment, which often is to become drug-free. Staff discuss the reasonableness of aiming for that goal when the treatment period is relatively short:

… then you kind of have to help the patient … to break it down to … “what is it that I need help with right now here to be able to reduce my use, or maybe even be completely tapered off drugs during these weeks”. (Staff T)

Even though patients express that staff lacks understanding of what it is like to live with addiction problems, they say the staff know “their stuff”, referring to theoretical knowledge about addiction, and they let the staff make most of their treatment-related decisions.

Tailoring schedules is a fundamental part of the treatment process, and several types of choices appear in this arena. The patient and the staff tailor a schedule for phasing out the drugs and a schedule for interventions that the patient is expected to attend during the treatment. Patients make “menu choices” when offered a choice among available options, and “delegated choices” when they ask the staff to propose schedules and interventions (i.e., due to uncertainty about the best options). When patients express special preferences (e.g., a wish to participate in interventions they are familiar with from previous treatments), they make “considered choices”. This can also be a “negotiated choice”, where staff and patients together discuss alternatives that are acceptable to both parties and include healthcare regulations.

Patients express that schedules are devised according to their wishes (e.g., when someone needs to leave and pick up children at preschool at a certain time or because they have to care for a sick relative at home). This wish to accommodate other responsibilities in the patients’ everyday life in the schedules leads both parties to make “negotiated choices”.

During staff conferences, they discuss patients’ treatments and schedules and sometimes make choices based on patients’ wishes and discussions with other professions, leading to a “considered choice” by staff:

… they can't do everything, and don't need everything … so now you have to think about how much they need to be somewhere other than at home, what is the reason, and which groups do we think are helpful, what do they think is helpful, and then try to sync this into the schedule, because this is a puzzle for them. (Staff U)

A tension may here be found between how patients and staff set their priorities. For the patients’, considered choice in this arena is about the structure, while staffs’ considered choice concerns the content of suggested measures relevant to the treatment aim.

### Obligatory Interventions

Patients at the DCU are expected to participate in certain mandatory interventions, individual and group based. Patients also have planned individual meetings with psychiatrists and other professionals at the DCU. Despite mandatory commitments, staff often need to rearrange meetings and activities temporarily due to staff sickness absence, making “spontaneous choices”. Sometimes the patient's care coordinator is not present, and the patient must then accept guidance from another staff member, also making a “spontaneous choice”*.* In another specific situation, staff need to make “spontaneous choices” when patients’ participation is not followed, leading to a “considered choice” to discharge the patient from treatment and an “either-or choice” for the patient.

The group interventions that patients are offered are evidence-based and recommended in other areas and types of clinics but adapted for patients with substance abuse undergoing treatment in day care to varying degrees. The staff make “considered choices” when adapting interventions. When participating in an intervention, patients report that they can choose to be committed during sessions or not, putting them into situations where they have to make “spontaneous choices” on every occasion:

A lot is what you make of it; you just sit out the time: ‘finally it's been an hour now it's over’. But if you get a little involved in it, you always get something out of it, but then it's a lot of fluff and it could have been a little more, but it's not so easy either … (Patient AB)

Patients appreciate that the staff are caring and want them to feel good while attending mandatory treatments at the DCU. The staff are perceived as accommodating, with a desire to let everyone have a say during courses and treatments at the DCU. Patients are encouraged to explain how they experience their situation and their addiction, but they are also asked questions about how they perceive the courses and the treatment. One patient said:

you usually feel like a “jerk”, but here you get such good treatment. (Patient D)

In addition to their interactions with health care, patients often have planned or mandatory contacts with other authorities. Based on the patient's situation, the patient and staff meet other authorities to plan and try to coordinate available efforts to support the individual in the process and choice of becoming drug-free. Here, patients are often seen to end up in a “hindered choice” when regulations clash or resources are limited or cannot be made available to the patient at the appropriate time.

### Non-obligatory Interventions

Between scheduled interventions, patients can make “spontaneous choices” when participating in other activities and meeting others (both patients and staff) in the DCU. They can have lunch together, drink coffee, read newspapers, play boardgames, or chat:

… a lot of the time, you don't have to do much, but just be here. You calm down, slow down and a lot of stress and stuff goes away … (Patient D)

Patients express that choosing to participate in non-obligatory activities gives them sober and meaningful options. Frustration was expressed because there were fewer options and choices as a result of COVID-19 restrictions. Staff expressed that attending the DCU gives patients structure in everyday life, and patients said that they appreciated the routines. Patients also expressed that it feels good to “get away” from their former everyday life involving drugs. Several patients addressed the dilemma that routines are only there when attending treatment, that they must create routines on their own on evenings and weekends, and later when they exit day care. Even though patients were free to engage in activities on their own at the DCU, individually or together with other patients, they primarily make “delegated choices” to participate in the non-obligatory interventions led by the staff.

Peer support workers (PSWs) are on site at the DCU and are invited to group discussions. PSWs have a special role because they meet patients but do not participate in all staff meetings. They are appreciated by patients because they share their experiences and patients often choose to partake in interventions with PSW:

There is no one else who knows more than another addict; they are worth their weight in gold. They have been in the same situation and are safe now in their sobriety. (Patient AB)

This is not an obligatory part of the treatment, therefore patients make “spontaneous choices” every time they decide to talk to a PSW and share their lived experience.

During group discussions, patients are perceived as more open and honest and choose to talk more about their situation than they do in direct contact with other staff. PSWs are guided by the concept of Narcotics Anonymous: “the only way is to be completely drug-free”, giving patients an “either-or choice”. PSWs have no pre-determined schedule for who is attending the meetings and therefore make “spontaneous choices” when asking straightforward and sometimes tough questions and inviting patients to answer in-depth follow-up questions. At the same time, PSWs make “considered choices” when relating to previous experiences:

Well, you have to be able to meet people in their chaos, a little bit, I mean it's easy for me, I can sit down and talk to anyone really, and that's because I've been in there myself, wishing someone would come to me. And maybe they need to hear others express things that they can't put into words. (Peer support worker KA)

The “spontaneous choices” made during the non-obligatory interventions are considered to lead to positive patient experiences, but these situations are rarely recognized as a basis for further treatment options.

### Discharge

When patients and staff meet to plan discharge in collaboration with other stakeholders, the patients’ choices do not always correspond to organizational rules and guidelines. Patients express that they rarely feel involved in post-treatment decision-making and that their choices do not matter because final decisions are made by others, indicating a wish for a “negotiated choice”. Both patients and staff express how patients often end up falling through the cracks when cooperation between health care and other authorities fail. In that situation, both patients and staff are left with “hindered choices” when making plans for exiting the DCU, limited by rules and guidelines. When accepting an eligible option, patients often make an “either-or choice” because these choices are conditional due to regulations from different stakeholders. This is exemplified by a patient who was not allowed to keep his private apartment during his stay at a treatment home, although both the patient and the probation service emphasize the importance of having a place to stay after discharge.

In addition, patients, staff, and PSWs request a clearer connection to the outside world, outside the “thick walls of the DCU” (i.e., the patients’ everyday life and the reality that they will face after exiting). Staff and PSWs propose that patients make “delegated choices”; they suggest that they join Narcotics Anonymous, non-governmental organizations, and/or contact municipal interventions on their own after leaving the DCU. To facilitate “considered choices”, both patients and PSWs ask for visits to treatment homes or dialogue meetings with different authorities.

One patient explained that “when you have quit and are discharged from here you are far from finished you don't feel good when the support is finished” (patient AB), emphasizing the need to completely change one's social context to succeed. Similarly, another patient stressed the importance of having a clear plan after discharge:

It would have helped a lot if there had been a clear plan for the time afterward, if you had not come home to an empty apartment without having anything to do. Otherwise, you go back to the same routines, the same friends, and doing the same things as you did before. (Patient C)

To establish a drug-free life after discharge, patients must maintain the newly established routines and find new friends and activities. This includes having the ability to make “considered choices” and “spontaneous choices” several times in their everyday life after exiting.

## Discussion

Supporting individuals with addiction to making choices during DCU treatment was multifaceted and appeared in different forms and situations during the treatment process. Seeking help represented part of a choice process aimed at a drug-free life, yet external factors, such as organizational structures, influence what, when and how choices could be expressed. While most choices involved both patients and professionals, considered choices were more frequent among staff, whereas menu and either-or choices primarily involved patients.

Patients reported challenges in obtaining the support and healthcare services they needed to manage their addiction and taper their drug use. Healthcare professionals, in contrast, emphasized that initiating contact with patients did not fall within their professional responsibilities, and instead referred patients to the clinic's website where contact information for the DCU was available. Furthermore, the findings suggested that patients tended to delegate to staff the authority to determine the content of their treatment but, on the other hand, expressed a need for extended support to manage their everyday life after the period of treatment.

Both the hindered choices for patients in the inquiry arena and the delegated choices regarding treatment content in the intervention arenas can be examined in relation to the concept of health literacy. Health literacy is defined as the ability to make informed health decisions in the context of everyday life, encompassing personal, social and environmental settings (Kendir & Breton, 2020). Studies have shown that patients with substance use disorders have lower health literacy compared to other treatment-seeking or general population groups (Dahlman et al., 2020; Degan et al., 2022). As health information needs vary according to age, health status and social and environmental conditions, health literacy should be understood as a context-dependent and dynamic construct rather than a static individual attribute. Individuals’ abilities to access, understand and apply health information are shaped through interactions with multiple actors and systems, including healthcare professionals and organizations. Lower health literacy has been associated with unemployment and lower education (Dahlman et al., 2020), which may contribute to patients with substance use disorders deferring important decisions to professionals, struggling to participate in treatment discussions and lacking the knowledge needed to make voluntary choices. Consistent with this, Andersson and Johnson (2020) emphasized that patients’ knowledge of available options is crucial for gaining influence over their treatment and finding the strength to make meaningful changes. In our study, however, deliberately leaving decisions to professionals was understood as an expression of trust in their expertise and satisfaction with the care received. While deferring to professionals can reflect confidence in expertise, it also highlights a potential gap between treatment practices and the skills patients need to live independently and maintain a drug-free life post-treatment.

Thus, the results have revealed a gap between the structure of measures during treatment and what is needed to live an independent everyday life without drugs when the treatment is over. This gap could be discussed in relation to the concept of recovery capital. Studies have highlighted the importance of improved social and personal functioning and not solely control of substance use (Cano et al., 2017; Vanderplasschen et al., 2019). Rosic et al. (2021) showed that goals related to patient functioning after leaving treatment were significantly associated with becoming drug-free. Not having the knowledge or motivation for making considered choices could also be discussed from occupational science and everyday life perspectives. This can be exemplified by Hammell (2020) who challenged the belief of individual free will. In this sense, choice as well as health behaviors and actions should be seen as a result of unequal distribution of social conditions and structures that influence people's abilities to make relevant choices. From this perspective, examining choices in isolation from the overall life situation of people with substance use disorders risks providing a narrow view. Focusing specifically on choices offers valuable insights into recovery-related processes but may overlook broader social, economic, and personal contexts. While this study focuses on choices, these are embedded within wider life circumstances that shape recovery. As Robertson and Nesvåg (2018) emphasized, transitioning to a non-using lifestyle involves profound changes in daily practices, relationships and social networks.

Common concepts used in the context of addiction treatment are motivation and self-efficacy (SAMHSA/CSAT Treatment Improvement Protocols, 2019). Even so, the results showed a gap between such intentions and patients’ worries about managing a drugfree life after discharge. Aims of treatment were to enhance the patient's motivation to reach the desired goals and to support them to improve their sense of self-efficacy and make relevant choices during treatment. Bandura (1977) defined self-efficacy as the belief in one's ability to carry out actions needed to manage challenges, shaping whether individuals initiate, sustain and persist in coping behaviours. Fostering self-efficacy bolster resilience, recovery adherence and relapse prevention and is found linked to better substance abuse treatment outcomes, but it has had little influence on treatment design and further research is needed to understand its role and develop effective ways to enhance it (Kadden & Litt, 2011).

The ability to make choices can also be discussed from a philosophical perspective. Racine & Rousseau-Lesage (2017) proposed a dynamic view for understanding choices and free will. In this sense, the ability to make choices constantly changes depending on prerequisites within the person with addiction and the context of treatment. A dynamic understanding of how the ability to make choices changes over time is exemplified by the finding in our study whereby patients talk about making spontaneous choices during participation in obligatory or non-obligatory interventions. While staff demonstrated consideration of patients’ needs, this may also suggest a gap between the two parties. Increasing awareness of how patients’ positive experiences relate to treatment, and incorporating these into discussions about treatment plans, could empower patients to make more informed choices that are better aligned with their individual needs both during and after the treatment period.

Mostly, hindered choices were found before and at the end of treatment due to misaligned regulations or external measures. The impeding factors were described as emanating from regulations and measures from other stakeholders, not synchronized with further need for support that occurred during the treatment. Further elaboration of possibilities for cooperation between stakeholders in a more recovery-oriented model has been suggested as one solution (Ashford et al., 2020). Further research is needed to explore patients’ opportunities to influence choices and bridge the gap between treatment and everyday life after discharge (Friedrichs et al., 2016; McKay et al., 2015).

To summarize, the results show a dual standpoint: (1) patients’ experiences of choice and scope for action and (2) staff's work practices in which choices are produced and realized. From patients’ accounts, choices are experienced as something negotiated in interaction with the unit and other actors, which alternatives become possible shifts across time and situation. From the vantage point of staff's work practices, choices are produced through local routines, templates and opportunities for decision-making; here, alternatives are translated into what is operationally feasible. The comparison shows that what appears in patients’ experiences as individual choices is, in practice, shaped by ruling processes from assessment, meeting templates and documentation. Rather than treating choices as mere preferences, the result demonstrates how alternatives arise and are narrowed within concrete workflows. The results suggest that prioritizing patient choice could increase engagement in treatment planning and foster self-efficacy in sustaining a drug-free lifestyle. Integrating health literacy initiatives within the treatment process may further enhance patients’ ability to make informed decisions, with guidance and follow-up helping them navigate their options.

### Methodological Considerations

The choice of method proved to be appropriate for this study because it provided an opportunity to investigate the research questions and increased understanding of the choices made by the patients and the staff during treatment for substance use disorder. Our contribution lies in empirically demonstrating how choices were organized rather than merely articulated: they took shape within text-and-rule-mediated practices in everyday work. This aligns with research that reconceptualizes choices as institutionally configured (Campbell & Gregor, 2008; Mykhalovskiy et al., 2020), while our analysis remains firmly grounded in content analysis based on interviews and observations.

The timeframe for the data collection was 7 weeks of field studies, which can be considered sufficient to get insight into the different sources of material. Seven weeks also coincide with an average treatment period, supporting the plan to cover the entire treatment process. COVID-19-related restrictions resulted in fewer patients participating simultaneously in the DCU, which in turn limited the opportunities for observation. To compensate for these limitations, additional interviews and observations were conducted.

The topic of the study may be considered sensitive, which requires extra care in relation to the participants. An uneven balance of power must also be considered. Therefore, it is particularly important to protect the participants, especially the patients, from being identified and potentially harmed. We believe that the way in which the selection of interviewees in the study was made and de-identification of the quotes ensure that the participants cannot be traced and that their anonymity and integrity are thus protected.

Triangulation of interviews, field observations and document reviews provided multiple perspectives on choices. Researcher triangulation and staff feedback enhanced trustworthiness. Our study also aligns with contemporary quality criteria for qualitative research as outlined by Hellman (2025). Reflexivity was applied throughout the research process, with researchers reflecting on positionality and potential biases. Transparency was ensured through detailed documentation of study procedures, and contextual sensitivity was maintained by situating findings within the organizational and social setting of the DCU. These principles support credibility, trustworthiness and relevance of the findings.

## Conclusions

This study highlighted choices made by patients and staff during addiction treatment. Patients’ opportunities for choice were limited and shaped by staff routines, organizational procedures and treatment policies, while support for sustaining choices post-treatment was often insufficient. The findings suggest that emphasizing patient choice could enhance participation in treatment planning and support self-efficacy in maintaining a drug-free life. Supporting autonomy may require guidance and follow-up and staff awareness of patients’ choices could inform and adapt treatment routines. Integrating health literacy initiatives during treatment may further strengthen patients’ capacity for informed decision-making. Future research is recommended to investigate the gap between patients’ opportunities for choice and treatment policies and routines.
